# Empowering Gliadin Detection: A Visible-Code Semiquantitative Lateral Flow System for Rapid and Reliable Results

**DOI:** 10.1021/acs.jafc.5c07872

**Published:** 2025-09-11

**Authors:** Wen-Hao Chen, Jill Christiansen Smith, Seaton Smith, Hui-Yin Huang, Chester Yuh-Cherng Chu, Yuen-Yee Choi, Yuyu Chen, Huan-Chi Chang, Chuan-Chih Hsu, Yu-Cheng Hsiao

**Affiliations:** † Research and Development Group, Leo Verification System Inc., Powell, Wyoming 82435, United States; ‡ Research and Development Group, Bion Inc., Taipei 110, Taiwan; § Department of Surgery, College of Medicine, 38032 Taipei Medical University, Taipei 110, Taiwan; ∥ Department of Surgery, Taipei Medical University Hospital, Taipei 110, Taiwan; ⊥ uMeal Co., Ltd., Taipei 110, Taiwan; # Department of semiconductor Engineering, Lung-hwa University of Science and Technology, Taoyuan 333, Taiwan

**Keywords:** gluten tester, gliadin tester, gluten detection, gluten tester, gluten sensor, IoT of image analysis

## Abstract

Wheat is a global staple, but its main protein, “gluten,” can trigger severe reactions in people with celiac disease, an autoimmune disorder affecting around 1% of the population. The only treatment is a lifelong gluten-free diet. Gliadin, a gluten component, is the primary trigger and is difficult to detect due to its low solubility. This makes it challenging for celiac patients to safely eat out or to enjoy diverse foods. To address this, we developed LEO (Lateral flow Enhanced by Optical imaging), a portable gluten detection system combining lateral flow assays with smartphone-based image analysis. LEO uses IoT-enhanced technology to deliver results in under 3 min, accurately quantifying gliadin with over 98% accuracy and sensitivity below the FDA’s 20 ppm threshold. In real-world trials, LEO successfully detected hidden gluten in mislabeled “gluten-free” restaurant dishes. With its fast, accurate results and user-friendly design, LEO supports safer food choices for individuals with celiac disease and is suitable for personal, clinical, industrial, and regulatory use.

## Graphical abstract

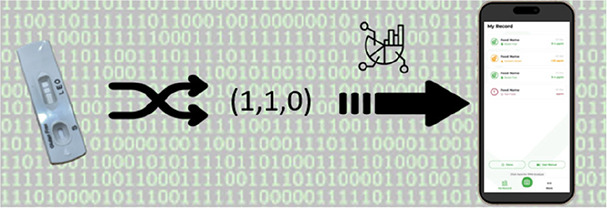

## Introduction

According to data from the U.S. Food and Drug Administration (FDA), there are nine common food allergens globally: wheat, peanuts, tree nuts, milk, eggs, soy, fish, sesame, and shellfish.^1^ Reported symptom prevalence indicates that approximately 8% of children currently suffer from food allergies,^2^ while the prevalence among adults is around 13%.^3^ It is estimated that annual healthcare costs associated with food allergies amount to approximately $25 billion.^4^

Since the onset of the COVID-19 pandemic, food prices have increased by 10–44%, depending on the region and type of food.^5,6^ Consequently, global food insecurity rates have surged regardless of individuals’ allergy status.^7–9^ Among populations with food allergies, instances of food insecurity are believed to be more prevalent than in the general population.^10–12^ For some patients and families, addressing food insecurity represents a critical area of discussion.

Despite the implementation of measures to reduce allergen exposure, incidents of cross-contamination and mislabeling continue to occur frequently in restaurants and food production facilities.^13–15^ Wheat allergy is one of the most common and well-known food allergies, clinically categorized into celiac disease^16–19^ and nonceliac gluten sensitivity (NCGS).^20–22^ The primary protein responsible for allergic reactions is gliadin. Since gliadin is insoluble in water^23^ and readily forms fibrous precipitates with glutenin,^24^ cross-contamination is particularly prone to occur during food processing or handling, posing significant health risks to individuals with gluten allergies.

Although numerous studies have focused on developing gluten detection platforms using techniques such as enzyme-linked immunosorbent assay (ELISA),^25^ surface plasmon resonance (SPR),^26^ and electrochemical sensors,^26^ these methods often face barriers to market acceptance due to their high costs and complexity. Consequently, there is an increasing demand for reliable and accurate gluten detection methods.

Lateral flow assay (LFA) is a well-established, convenient, user-friendly, and portable biochemical detection technology widely applied in various fields, including pesticide detection, cancer screening, food allergen testing, and genetic testing.^27–29^ However, current LFAs are limited to qualitative testing, which results in poor accuracy and a high incidence of false negatives and positives (Hook effect), restricting their practical applications. This study aims to develop a next-generation LEO Code Quantify Assay System by integrating competitive and sandwich detection methods into a single strip format within the LFA framework. In the LEO code quantify assay system, we integrated the hardware of the lateral flow and software of the image assay system to identify the quantity of gluten in food. Using this system, in this work, we integrate both the competitive and sandwich assay formats onto a single test strip. By assigning different detection sensitivities to the competitive and sandwich regions, we establish a defined detection range, a model that has not been reported before. Based on this, we hope every gluten-intolerant person can enjoy the food and **L**ove **E**ating **O**ut (LEO) again. For such a consideration, we designed the O line (operation line as the C control line), E line (Eating line, with a sensitivity of 5 ppm of gluten; competition model), and L line operation line as the T Test line (sensitivity is 10 ppm and hook effect at 20 ppm; sandwich model) in one strip. Based on this model, we can quantify the gluten content in the sample with the E line and double-check using the “L” line (shown in Figure 1A). The strip result is shown in Figure 1B. This design can powerfully increase the accuracy of the assay system. Following previous research,^27^ we used an ionic liquid for a quick extraction of gliadin in the sample and successfully reduced the time of testing to 3 min. All of the testing results can be uploaded to the cloud using a person’s phone and the LEOMyFood app image analysis system; depending on the image assay, the detection limit can be raised to 0.1 ppm of gluten. The user can get results from the app in 1 min and document the testing results by date, location, and share with others (shown in Figure 1C).

**Figure 1:**
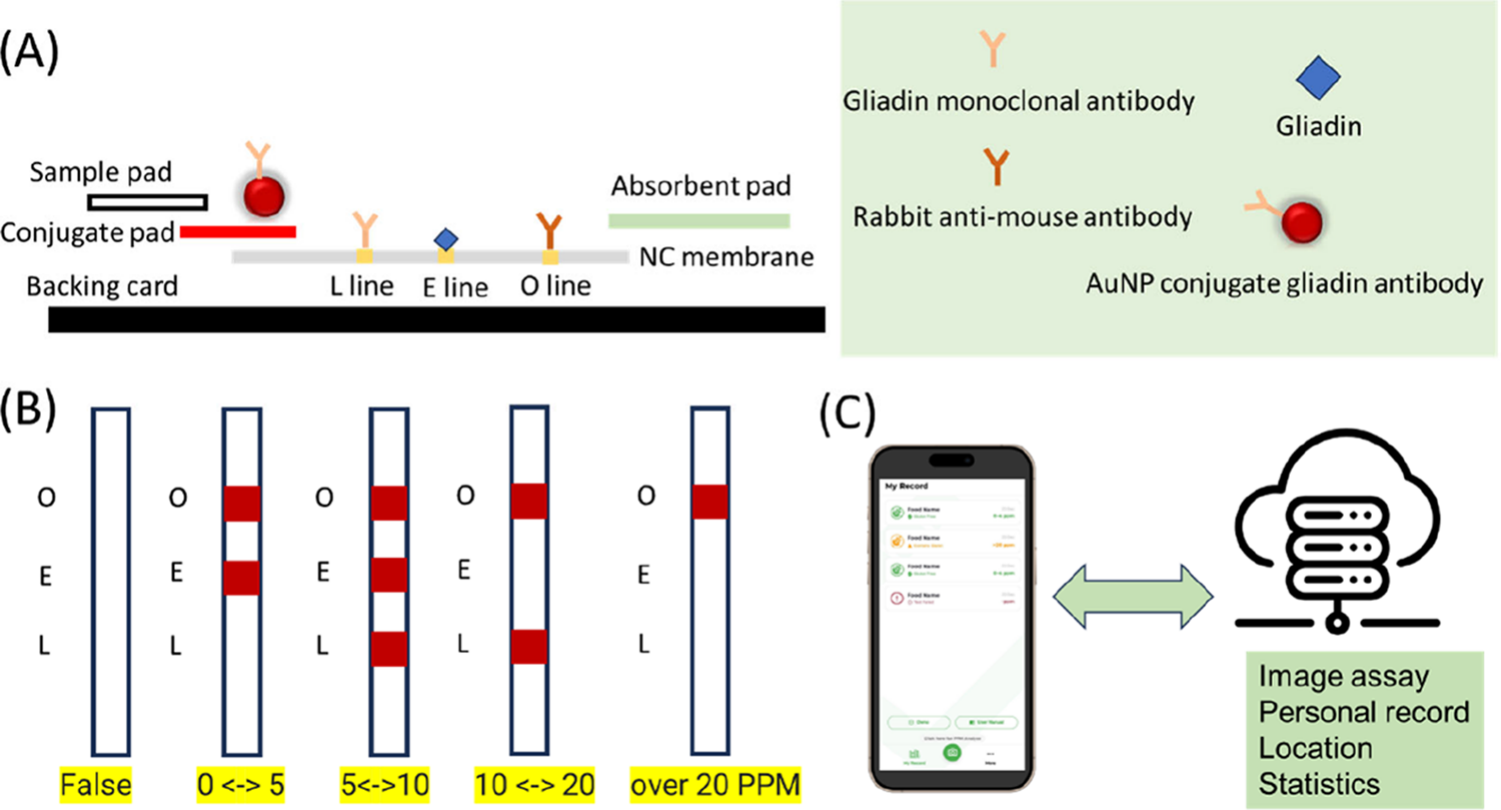
(A) Quantity lateral flow model structure. (B) Code system applied to a new lateral flow model. (C) IoT of the code system for the quantity lateral flow system. Data can be updated to a cloud-based platform for gluten content analysis, and users can capture and upload images of the test strip through their smartphones.

## Results and Discussion

### LEO Code Quantifying Assay System

In the new model of the LEO code quantifying assay system, we combine testing results from previous technology and the sandwich model of the lateral flow into one sensor strip. In this new model, we have designed the conjugated pad as AuNPs conjugated with a gliadin antibody (AuNP-Ab) and designed the LEO lines on the Nitrocellulose membrane (NC membrane). On the NC membrane, we coated rabbit anti-mouse IgG on the O line. The rabbit anti-mouse IgG can measure the antibody if it is workable on this NC membrane, just like the function of the Control line. The gliadin was coated on the E line. (The gliadin antibody was from the outbred mice immunization, and the secondary antibody is goat anti-mouse IgG (purchased from Abcam.))

Here, there is a competition model used for the gliadin assay. When the gliadin concentration is high enough for full binding with AuNP-Ab, the AuNP-Ab cannot bind with gliadin on the E line. There will be no signal on the E line. The mouse anti-gliadin antibody was coated on the L line. The L line used the sandwich model for gliadin detection; the L line will be dark when the sample has gluten, and the hook effect will happen when gluten results are higher than 20 ppm. The concentration of gliadin and mouse anti-gliadin antibody should accurately coat the NC membrane (shown in Figure 1A). Usually, the sandwich model will have a hook effect in a high concentration of the target protein. The hook effect will induce a false negative result and put the user in a dangerous situation. In the LEO code quantifying system, we have solved this issue. The E line is used to measure the 10 ppm of gluten detection. The sensitivity of the L line is 5 ppm of gluten, and the hook effect will show in a gluten concentration higher than 20 ppm. The code we designed will follow the list of O, E, and L and signal by light or dark (defining the light as 0 and dark as 1 for testing results). Based on this design, 0–5 ppm of gluten will show in (1,1,0), 5–10 ppm is (1,1,1), 10–20 ppm is (1,0,1), and over 20 ppm is (1,0,0). A failed test will show (0,0,0), (0,1,0), (0,0,1), or (0,1,1) (shown in Figure 1B). The user can use our app for image analysis, record testing results, and share results with other people (shown in Figure 1C).

### Characterization of AuNPs and Antibody Conjugated with AuNPs

AuNPs exhibit a unique local surface plasmon resonance (LSPR) effect that is sensitive to particle size and surface chemistry,^30,31^ Gold nanoparticles are characterized by distinctive surface plasmon resonance (SPR) properties. Modifications to the nanoparticle surface, including the incorporation of biorecognition elements, induce alterations in surface charge, which commonly lead to a blue shift in the SPR wavelength.^32–34^ Accordingly, the proposed LEO leverages this effect through the use of citrate-reduced AuNPs. We synthesized AuNPs through several steps: nucleation (reduction of HAuCl4 to Au atoms),^35,36^ followed by the growth and agglomeration of these atoms into nanoclusters, resulting in a red AuNP solution. The TEM image and ultraviolet–visible (UV–VIS) light spectrophotometry are shown in Figure 2A,B. The average size of AuNPs is 11.62 nm (shown in Figure 2C).

**Figure 2:**
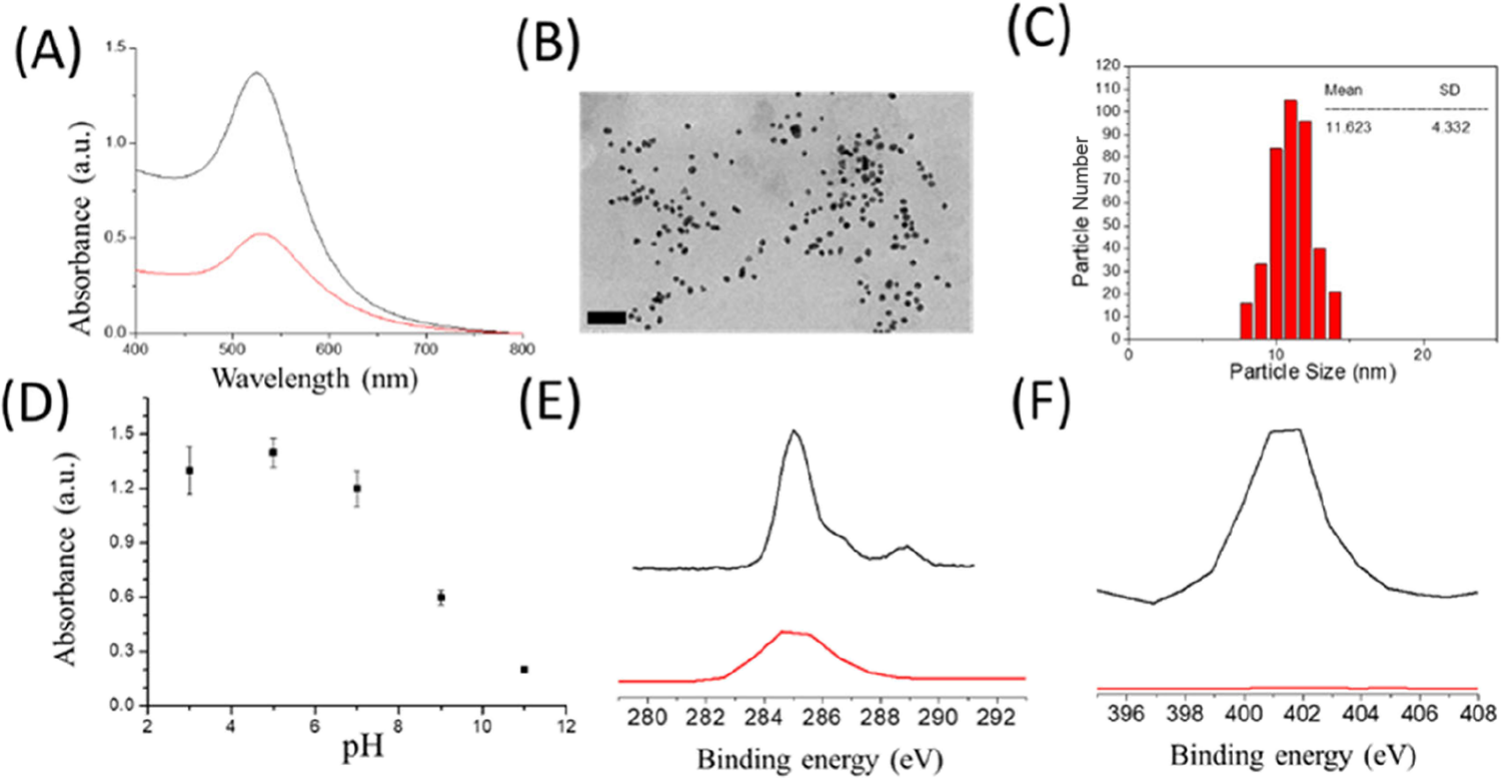
(A) UV–vis spectrum of AuNPs (Black) and AuNPs conjugated with antibody (red). (B) TEM image of Au particles. (Scale bar is 100 nm). (C) Size distribution of AuNPs based on diameter measurements. (D) UV–vis absorbance of the antibody conjugated with AuNPs under different pH solutions. (E) XPS spectrum of C 1s in AuNPs with (black)/without (red) antibody coating. (F) XPS spectrum of N 1s in AuNPs with (black)/without (red) antibody coating.

### Optimal Antibody Concentrations for Conjugation with AuNPs

The pH value influences the conjugation of antibodies to AuNPs. We investigated the effect of pH on the antibody–AuNP conjugation.^37,38^ Here, we tested the antibody conjugated with AuNPs in different pH solutions. The AuNPs can avoid the aggregation or color shift from the salt effect after successful conjugation with the antibody. When the AuNPs have significantly aggregated, they will induce the absorbance peak shift in the UV–vis spectrum. Based on this issue, we followed the peak of 520 nm absorbance in the UV–vis spectrum to see whether the AuNPs aggregated or not. In the test, we found that the AuNPs conjugated with the antibody stably under the pH 3, 5, and 7 solutions. The AuNPs were aggregated when the pH went higher than 7 (shown in Figure 2D).

The spectral results revealed a narrow absorption peak at 520 nm of AuNPs without antibody and a narrow absorption peak at 523 nm of AuNPs conjugated with antibody (shown in Figure 2A (red)). The peak shift of AuNPs in UV–visible spectra showed that the antibody was coated with the AuNPs. In this report, we also use DLS to measure the zeta potentials of AuNPs and AuNPs conjugated with an antibody. The data are shown in Figure S1.

### Characterized Antibody Conjugated with AuNP By X-ray Photoelectron Spectroscopy (XPS)

XPS is a popular tool for measuring the atomic state on a substrate surface.^39–41^ Specifically, it can rapidly determine the atom and oxidation states on a substrate for measuring surface modification. In this study, the AuNPs and AuNP-Ab were dropped on a glass plate and dried for 48 h to prepare the XPS sample. XPS C­(1s) (Figure 2E) and N (1s) (Figure 2F) spectra were used to characterize AuNPs with/without antibody coating. In the XPS C (1s) spectra, there is a strong signal at 284.5 eV from the C–C bond of AuNPs with (Black) /without (red) antibody coating.^42^ In the case of AuNPs-Ab, there are significant signals at 286.3 and 288.5 eV from C=O and N–C=O bonds. This special signal is from the peptide bond of the antibody^43,44^ and proves that the antibody was being coated on AuNPs.

Figure 2F shows the XPS N (1s) spectra result of AuNPs (Red) and AuNP-Ab (Black). In N (1s) spectra, there are significantly different signals at 402 eV of AuNPs and AuNP-Ab. The 402 eV signal of AuNP-Ab is from the amide bond of the peptide.^45^ The amide bond is a special signal of the antibody (black line). The AuNPs without the antibody coating do not have the 402 eV signal (red line).

### Sensitivity of LEO

Using the LEO quantifying code system for quantifying the content of gluten, we developed an image analysis system to measure the sensitivity and stability of gluten testing results. This system facilitates the evaluation of the analytical performance of the proposed LEO for rapid gluten detection. First, the response curves were generated by varying the gliadin concentrations. These curves were then incorporated into the LEO image analysis system as lookup tables, which enable quantitative analysis. The LEO analysis demonstrated high sensitivity, precision, and quantitative accuracy. To assess the sensitivity of the LEO, we tested eight gluten concentrations (0, 3, 6, 9, 12, 25, 30, and 40 ppm) in triplicate. From the test result, we could find a dark signal of the E line from 0 to 9 ppm, and the signal became lighter following the gliadin content rise. For the L line, a visible black line appeared along the test line as the gluten concentration was increased from 9 to 12 ppm. The black color on the L line was reduced from 12 to 25 ppm; this is the hook effect in this strip^46–48^ (Figure 3A). Hook effect is from the sample content of too much gliadin and affects the antibody (on L line) binding with gliadin. This effect cannot be avoided, and the hook effect will increase the risk of system failure in high concentrations of gliadin. Furthermore, the color intensities change on the E and L line with the gluten content (Figure 3B). We used the following equation to calculate the detection limit of the LEO gluten quantify system: *S*_dl_ = *S*_reag_ + 3σ_reag_. In here, *S*_dl_ is the detection limit, *S*_reag_ is the signal of the reagent blank, and σ_reag_ is the standard deviation of the reagent blank. In this report, the calculation indicated the detection limit to be 10 ppm on the E line and 5 ppm on the L line. Moreover, the entire test procedure was completed within 3 min.

**Figure 3:**
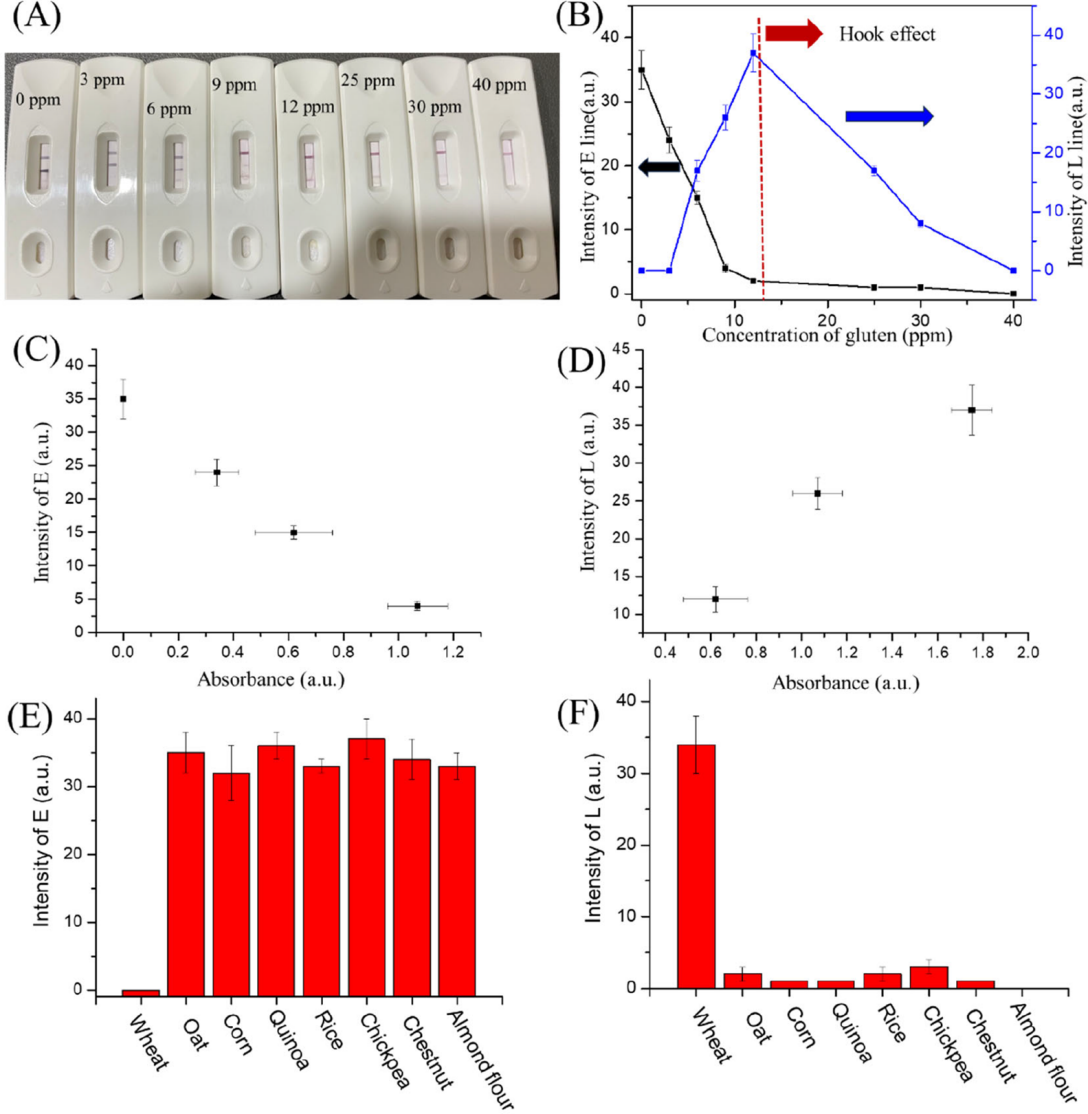
(A) LEO gluten test (left to right: 0, 3, 6, 9, 12, 25, 30, and 40 ppm) results. (B) Samples with varying doses of gliadin were analyzed in the S­(E) line (black) and T (L) line (blue) using the LEO, and response curves were generated. (C) LEO E line results exhibited an excellent match with the ELISA results (*R* ^2^ = 0.988). (D) LEO T­(L) line results exhibited an excellent match with the ELISA results (*R* ^2^ = 0.981). (E) S (E) line shows the specificity of the LEO for different flour samples. (F) T (L) line results from the specificity of the LEO for different flour samples.

### Analytical Performance of LEO Image Analysis

The limit of detection for gliadin was 10 ppm on the E line and 5 ppm on the L line, both of which are lower than the threshold dose for gluten allergens (20 ppm). For comparison, the same samples were also analyzed by using the traditional ELISA technique. The results from the LEO assay showed a strong correlation with those obtained from ELISA, with an *R* ^2^ value of 0.99 on the E line (Figure 3C, comparing the range from 0 to 10 ppm) and an *R* ^2^ value of 0.983 on the L line (Figure 3D, comparing the range from 5 to 20 ppm). Importantly, the LEO assay demonstrated a significant advantage in terms of speed, providing results in less than 3 min, in contrast to the ELISA method, which took approximately 3 h to complete.

### Specificity of LEO

Specificity is a critical parameter for biosensors. To assess the specificity of the proposed LEO system, we tested flour samples from a variety of cereals, including wheat, oat, corn, quinoa, rice, chickpea, chestnut, and almond. Previous studies have indicated that milk could interfere with gluten testing, potentially causing false positives. In this study, we mixed each cereal sample with milk in a 1:100 ratio to simulate this potential issue. The results were quantified using image analysis, as shown in Figure 3E,F.

In the tests, a significant signal was observed—marked by a light line on the E line and a dark line on the L line—only for gluten-containing cereals, such as wheat. Notably, the black line for wheat flour appeared within 3 min, likely due to its high gluten concentration (Figure 3C). Each sample was tested six times under the same experimental conditions. These results demonstrate that the LEO gluten tester is capable of accurately detecting gluten, even in complex scenarios.

### Analytical Performance of LEO Quantifies Code System

The user-friendly design within the LEO rapid test is a quantifiable code assay system for analyzing test results (Figure 1B). In the code design, the threshold will be defined at 20 intensities for easy detection by the eyes. This system facilitates the evaluation of the analytical performance of the proposed LEO for rapid gluten detection.

In this test, we prepared the samples of 0, 6, 12, and 25 ppm of gluten (shown in Figure 4A) to quantify the intensity of the **L**imit-Line, **E**at-Line, and **O**peration-Line by image analysis. In the intensity analysis, the operation line (O line) has the same dark line under each different gluten content test. This proves that the total AuNPs-Ab is enough for each line absorbance (shown in Figure 4B). The intensity was reduced as the gluten content rose on the Eat line (E line). Based on the threshold definition, the E line was very light when the gluten content was higher than 10 ppm (shown in Figure 4C). On the Limit line, the signal was rising following the gluten content until 20 ppm of gluten, and reduced the intensity when the gluten content was higher than 20 ppm (shown in Figure 4D). This proved that the hook effect was significant when the gluten content was higher than 20 ppm.

**Figure 4:**
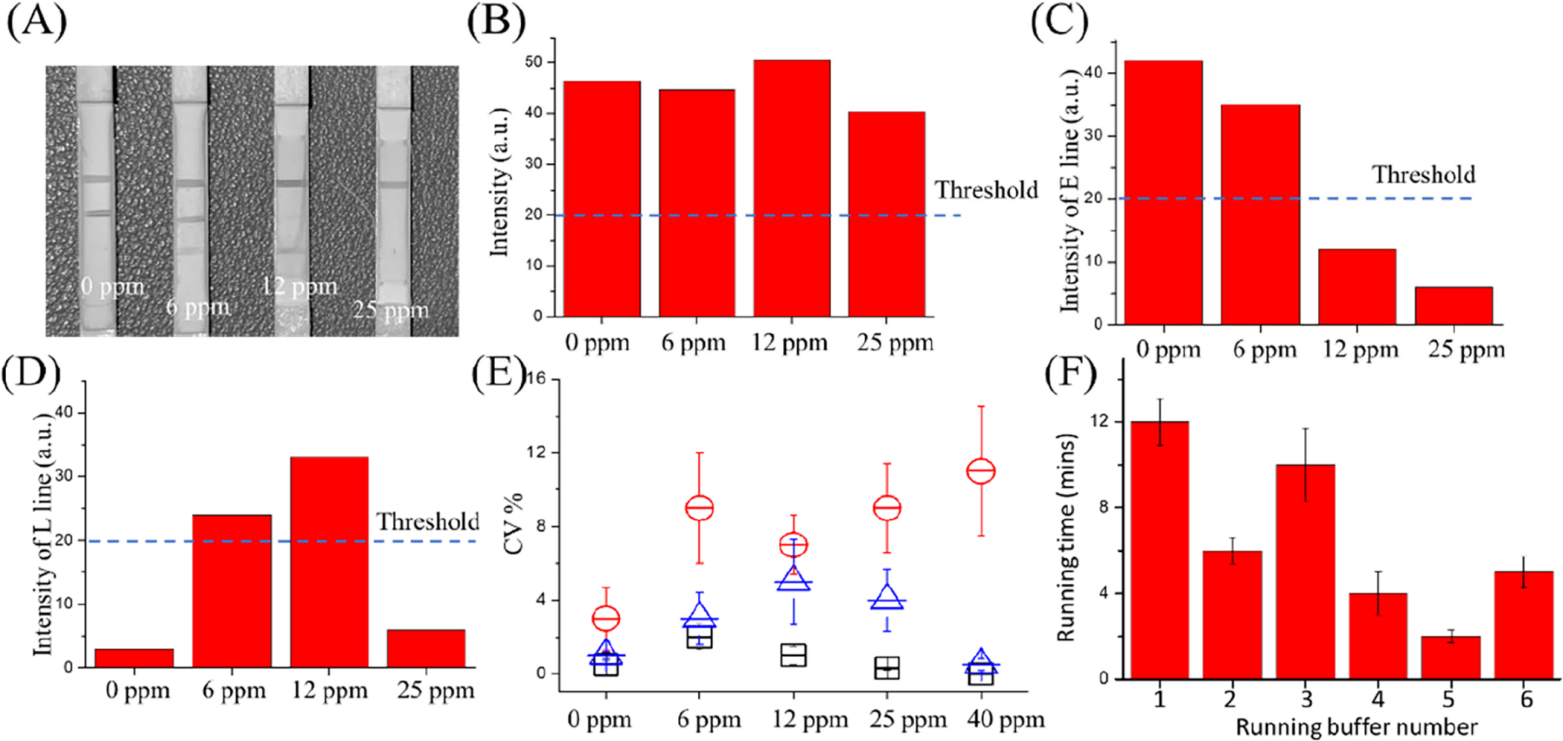
(A) LEO code quantify lateral flow system gluten test (0, 5, 10, and 20 ppm) result. (B) The image analysis of Operation line (C line) in different gluten content sample test. (C) Image analysis of Eat line (S line) in different gluten content sample test. (D) Image analysis of the limit line (T line) in different gluten content sample test. (E) LEO was noted to be highly reproducible. Both the intra-assay and the interassay variations were <4% of E line (Black) and <6% of L line (Blue). Intra-assay and the interassay variations of ELISA is less than 11%. (F) Time cost in testing by different running buffers. (1. Tris buffer only. 2. Tris + 0.5% TWIN-20. 3. Tris + 1% sucrose. 4. Tris + 0.5% TWIN-20 + 1% sucrose. 5. Tris + 0.5% TWIN-20 + 1% sucrose + 1% BSA + 0.1% IL. 6. Tris + 0.5% TWIN-20 + 1% sucrose + 1% BSA). Note: The threshold is the intensity of the result seen by the naked eye.

Intra-assay variability was evaluated by measuring eight replicates of five different standard concentrations (0, 6, 12, 25, and 40 ppm). The results demonstrated excellent intra-assay precision, with variations of less than 6% on the E line (Figure 4E, black) and less than 8% on the L line (Figure 3E, blue). Furthermore, intra-assay variations were evaluated and found to be less than 4%. Combining the C and L line for measuring the range of gluten level, the variations below 2% and intra- assay variations were evaluated and found to be less than 0.8%. In the intra-assay variations of the ELISA tester, the variations were below 12% (Figure 4E, red).

### Running Buffer Test

To investigate the time of LEO gluten testing, Tris buffer is known for its ability to enhance gliadin solubility.^47^ Thus, we used the Tris buffer as a base solution to prepare different running buffers to increase the speed of the gluten test (1. Tris buffer only. 2. Tris + 0.5% TWIN-20. 3. Tris + 1% sucrose. 4. Tris + 0.5% TWIN-20 + 1% sucrose. 5. Tris + 0.5% TWIN-20 + 1% Sucrose + 1% BSA + 0.1% [BDMIM]­[OMs]. 6. Tris + 0.5% TWIN-20 + 1% Sucrose + 1% BSA).

In the study, we found that the Tris buffer only has a longer testing time for gluten tests. The buffer 5 (Tris + 0.5% TWIN-20 + 1% Sucrose + 1% BSA + 0.1% [BDMIM]­[OMs]) reacts in the least amount of time for gluten testing. It only needs 1 min 20 s to start getting a visible result (shown in Figure 4F).

### Time Dependence of the LEO Gluten Test

Figure 5A shows the time dependence of LEO gluten testing with a buffer of 5 ppm. In this study, we tested 0 ppm of the sample to measure the time needed for the testing results. In the picture of Figure 5A, we found the visible line of E showed after 60 s, and the line became darker after 90 s. The clear results of the C line and O line were shown in 2 min. We also used the image analysis to measure the intensity of the results (shown in Figure 5B). The intensities of the O Line and the E line were raised following the time increase. In 60 s, the intensity of the E line is higher than the threshold. Comparing the O line, the E line is visible to the naked eye, but the intensity of the O line is still not dark enough for visibility. In less than 3 min, the intensity of the E and O lines was both dark enough and visible to the naked eye (shown in Figure 5B).

**Figure 5:**
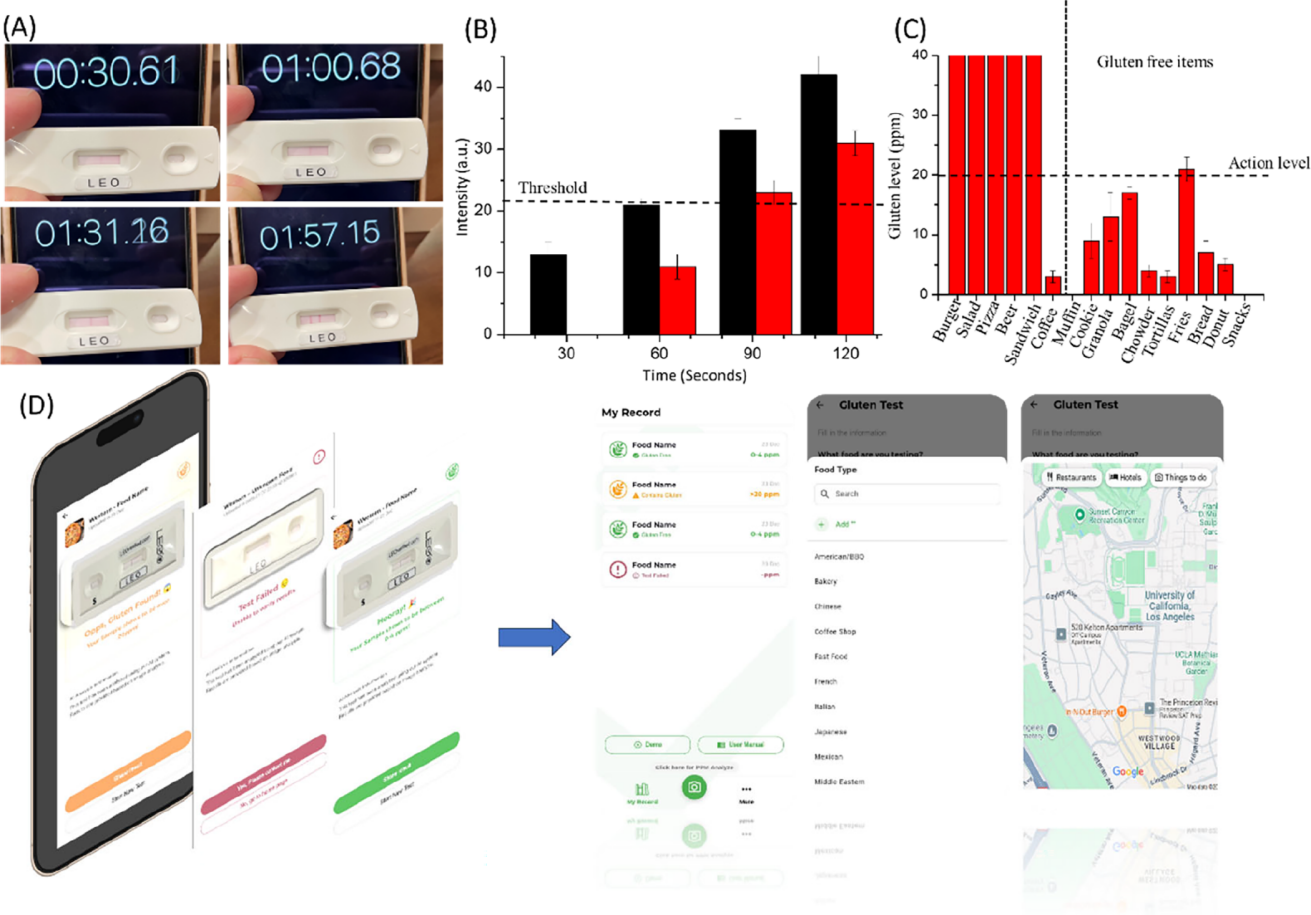
(A) LEO test result can be observed by the naked eye in 2 min. (B) Intensity results of E and L line in different time. (C) Food testing in restaurants with/without gluten free. (D) LEO code quantify app can analyze the gluten content in food, document the testing and share the testing result to others by a food list or food map. Note: the action level is 20 ppm of gluten based on the FDA definition.

### Gluten Recovery Rate with LEO

Recovery rate is an important metric for evaluating extraction systems, as it indicates the effectiveness of extracting a target substance from a sample. In this study, we assessed the gluten recovery performance of the LEO system.

Rice noodles, commonly known as gluten-free food, were selected as the sample for the recovery test. First, the rice noodles were tested for the absence of gliadin by using the ELISA method. Next, a 10 ppm gliadin solution was added to the rice noodles, and the solution was removed under vacuum to prepare a gliadin sample standard. The LEO system was then used to extract gliadin from this standard sample, and the gliadin concentration was measured using ELISA. The resulting data is presented in Table 1. A 100% recovery rate was defined as the recovery of 200 ppm of gliadin. After six repetitions of the recovery test were conducted using the LEO system, the average recovery rate was determined to be 100%.

**Table 1:** Recovery Test of LEO

	standard gluten (ppm)	extraction gluten (ppm)			recovery (%)
		E line	L line	average	
1	4	4.7	3.4	4.05	101.25
2	4	4.6	3.3	3.95	98.75
3	4	4.3	3.6	3.95	98.75
4	4	4.5	3.7	4.1	102.5
5	4	3.7	4.1	3.9	97.5
6	4	4.2	3.9	4.05	101.25
average	4	4.33	3.67	4	100
control	0	0	0	0	0

### Field Testing

In this report, we tested foods (burgers, salads with dressing, pizza, and beer) obtained from local restaurants by the LEO gluten tester. The profiling results (Figure 5C) showed the expected allergens, such as gluten in hamburgers and pizza, but we also detected unexpected antigens contributed by food processing. For example, the salads contained gluten from the salad dressing or from cross-contamination using a bowl to mix the salad that previously had croutons in it.

For the testing of food with a gluten-free label, as expected, products were largely devoid of the listed allergen. The special case is French fries. The fries have serious cross-contamination because the fryers may also be used by some products with gluten (i.e., chicken fingers). However, more than 95% of gluten-free products had a gluten content lower than 20 ppm.

Utilizing LEO’s interface with a smartphone, the App can analyze the result image and report the gluten level to the user. The App can trace personal dietary intake, recording antigen data with timestamps in a cloud server. As an example, we surveyed gluten-free menu items from seven local restaurants and logged the results (e.g., food name, gluten contents) with locale information. Among “gluten-free” items, we observed a wide spectrum of gluten levels (0–5, 5–10, 10–20, or >20 ppm). These results were then used to create an evidence-based restaurant map that can be shared online (Figure 5D).

### Testing of Commercially Available Products

We further evaluated the performance of the LEO system by testing various consumer food products, including packaged staples and desserts. Small samples (40 mg) were collected, and solid foods were crumbled before analysis. The performance of the LEO was compared with that of ELISA. The test results (Table 2) showed a high level of consistency between the LEO and ELISA. In some cases, the gliadin concentration was below the sensitivity threshold of ELISA (2 ppm), which could lead to missed detections. Overall, both LEO and ELISA demonstrated strong agreement.

**Table 2:** Food-Processing Test Using the LEO and ELISA

name	LFD system	ELISA
Ferrero	5–10 ppm	7 ± 2.2 ppm
Bagel	>40 ppm	>40 ppm
Fried Dumplings	>40 ppm	>40 ppm
Toast	>40 ppm	>40 ppm
Lay’s Stax Original Potato Crisps	0–5 ppm	0 ppm
Nestle Nesquik Chocolate Syrup	0–5 ppm	0 ppm
M&M’s Crispy Milk Chocolate Bar	5–10 ppm	3 ± 1.1 ppm
Campbell’s Chunky New England Clam Chowder	5–10 ppm	6.7 ± 2.5 ppm
Lindt Swiss Classic Milk Chocolate	0–5 ppm	3.4 ± 0.9 ppm
S&B Golden Curry Sauce with Vegetables Mild	5–10 ppm	10 ± 2.4 ppm
Hershey’s Cookies ‘N’ Creme Candy Bar	10–15 ppm	12 ± 3.2 ppm
Nestle Kit Kat Chunky Peanut Butter Chocolate	>40 ppm	>40 ppm
Barilla Capellini n.1	>40 ppm	>40 ppm
Lotus Biscoff Spread Crunchy	>40 ppm	>40 ppm
Munchy’s Oat Krunch Crackers Strawberry & Blackcurrant	>40 ppm	>40 ppm
Bento Squid Seafood Snack Original Thai Chilli Sauce	>40 ppm	>40 ppm

The lateral flow test for gliadin is a rapid and user-friendly assay method. The R-Biopharm gliadin test is the only strip-based assay for gliadin detection using the R5 antibody. The high sensitivity of the R-Biopharm test is attributed to the specificity of the R5 antibody. However, the R-Biopharm test still requires sample preparation, uses a buffer that is not shelf-stable (requiring refrigeration), and is time-consuming. Despite these limitations, the R-Biopharm gliadin test is widely used for gliadin detection.

In this study, we also compared the performance of the LEO system with that of the R-Biopharm gliadin test in food sample processing, and the results are presented in Table 3. The results revealed a high level of consistency between the LEO and the R-Biopharm test, confirming the effectiveness of the LEO system in detecting gliadin during food processing.

**Table 3:** Lateral Flow Test of Processing Food Using the LEO and R-Biopharm Gliadin Test

	label GF	LFD system	R-biopharm (R7003)
Guerrero Tostadas	yes	0–5 ppm	<5 ppm
Krusteaz GF All-purpose flour	yes	0–5 ppm	<5 ppm
Krusteaz GF Honey Cornbread mix	yes	0–5 ppm	<5 ppm
Sprouts GF Steel cut Oats	yes	0–5 ppm	<5 ppm
Pillsbury GF Choc Fudge Brownie Mix	yes	0–5 ppm	<5 ppm
Lay’s Stax Mesquite Barbecue Chips ChipsMcCormic	yes	0–5 ppm	<5 ppm
Kelloggs Multi grain club crackers	no	>40 ppm	>40 ppm
Smarties candy Bracelate	no	0–5 ppm	<5 ppm
Fritos Chili Cheese Chips	no	0–5 ppm	<5 ppm
Mild taco seasoning	no	0–5 ppm	<5 ppm
Cap’t Crunch Berries	no	0–5 ppm	<5 ppm

Additionally, by integrating the LEO with a smartphone app, we were able to track personal dietary intake and record gluten data with timestamps in a cloud server. This server documented the results along with local restaurant information. The data were then used to generate an evidence-based restaurant map, which can be shared online.

Food allergies represent a significant public health issue in the United States, with a substantial impact on both individuals and healthcare systems. Studies show that between 30% and 86% of allergic children suffer from multiple allergies, contributing to nearly 203,000 emergency department visits each year—90,000 of which are related to anaphylaxis. Gluten-related sensitivities, including celiac disease,^49–51^ nonceliac gluten sensitivity (NCGS),^52–54^ and gluten intolerance, affect about 5% of the population. Although a gluten-free diet is crucial for managing these conditions, its implementation can be challenging due to the frequent risk of cross-contamination. Ensuring proper diagnosis, accurately identifying food allergens, and enabling real-time monitoring are key steps in reducing the harmful effects of food allergies.

In response to these challenges, we developed the LEO, a point-of-care food testing system designed for accurate gluten detection. This sensor allows individuals to make informed dietary decisions and avoid unnecessary dietary restrictions. Compared to other gluten testers available, the LEO is more compact, faster, easier to use, and provides semiquantitative results (as illustrated in Figures S2 and S3). It also addresses the problem of false negative results caused by the hook effect through its unique quantifiable code system (Figure 1B). Moreover, the LEO is cost-effective, with assay costs under $10 per antigen test (Table S3) and does not require any additional equipment.

The LEO’s versatility and performance surpass other consumer-grade gluten detection methods by eliminating the need for complex pretreatment steps and multisolvent usage, while maintaining high specificity and sensitivity (Figure S5). Its small size and user-friendly design make it suitable for a wide range of applications, including consumer health protection, quality control, environmental monitoring, and supply chain management.

When compared to rapid tests and the ELISA technique, LEO offers notable benefits such as ease of use, accuracy, speed, and broader applicability (Table S2). These features make it an invaluable tool for a variety of food safety applications. Our goal is to expand the LEO platform to detect other common food allergens, such as peanuts, tree nuts, milk, and seafood, ultimately developing a comprehensive allergen detection panel. This sensor could be used to ensure food safety, verify the origins of food products, confirm the absence of contaminants, and support dietary restrictions for diverse needs.

Moreover, by modification of the affinity ligands, the LEO assay can be adapted to detect various other analytes, such as small molecules, toxins, and nucleic acids. This adaptability paves the way for applications beyond food testing, positioning the LEO as a powerful tool for a range of analytical purposes.

We believe that the portable LEO has the potential to revolutionize food analysis by providing more rigorous, evidence-based methods for consumer protection. It will help reduce accidental allergen exposure and aid in identifying issues within the food supply chain.

## Materials

### Reagents and Solvents

Hydrogen tetrachloroaurate trihydrate (HAuCl_4_·3H_2_O), wheat gluten, 1-propanol, methane sulfonyl chloride, imidazole, citric acid, Tris­(hydroxymethyl)­aminomethane (Tris) buffer, and phosphate-buffered saline (PBS) were obtained from Sigma-Aldrich (USA) or ACOS (Taiwan), as specified. Additional chemicals, including hydrochloric acid, sodium bicarbonate, sodium hydroxide, potassium chloride, sodium dihydrogen phosphate, disodium hydrogen phosphate, and sodium chloride, were also purchased from ACOS. Organic solvents such as dichloromethane, methanol, acetonitrile, and ethanol were acquired from Tedia (USA). Nitrocellulose membranes (Hi-Flow Plus 120), sample pads, conjugate pads, absorbent pads, and adhesive backing cards were obtained from Merck Millipore (Germany). Trisodium citrate dihydrate, bovine serum albumin (BSA), Tween-20, poly­(ethylene glycol) (PEG 20000), glucose, phosphate buffer solution (PBS), and 0.22 μm filters were supplied by Merck. Goat anti-mouse IgG was purchased from Abcam (UK). Deionized (DI) water used for all solutions and buffer preparations was purified by using a Milli-Q water purification system (Millipore, USA).

## Methods

### X-ray Photoelectron Spectroscopy (XPS)

The surface chemical states of gold nanoparticles were examined utilizing a Kratos Axis Ultra DLD spectrometer, which is outfitted with a monochromatic Mg/Al achromatic X-ray source functioning at 450 W in a high vacuum environment. Spectra were collected at a photoelectron takeoff angle of 45°. The binding energies were calibrated by referencing the C 1s peak of saturated hydrocarbons, set at 284.5 eV, as an internal standard.

### Enzyme-Linked Immunosorbent Assay (ELISA)

The quantification of gluten content in the samples was performed using a commercially available enzyme-linked immunosorbent assay (ELISA) kit (Crystal Chem, AOAC No. 011804). Sample preparation involved suspending 0.1 g of wheat-derived gliadin powder in 10 mL of 40% ethanol, followed by extraction for 5 min. The suspension was then centrifuged at 2500 × *g* for 10 min to remove particulate matter. The resulting supernatant was diluted 1:50 with a 1× diluent buffer. Prior to analysis, all samples were equilibrated to room temperature (20–25 °C) for 15 min.

During the assay, 100 μL aliquots of both samples and standards were dispensed in duplicate into wells of an antibody-coated ELISA plate and incubated at room temperature for 20 min. Subsequently, the wells underwent three washing cycles with 300 μL of wash buffer. Next, 100 μL of horseradish peroxidase (HRP)-conjugated secondary antibody was added to each well and incubated for an additional 20 min at room temperature. Following a second washing step, 100 μL of the tetramethylbenzidine (TMB) substrate was introduced and incubated for 20 min in the dark. The reaction was terminated by adding 100 μL of a stop solution, and absorbance was measured at 450 nm using a microplate reader. According to Codex Standard 118-1979, the gluten concentration is calculated as twice the measured gliadin content.

### Synthesis of Ionic Liquid

The synthesis of the ionic liquid was accomplished through an SN2 reaction involving 1-methanesulfonylpentane and 1,2-dimethylimidazole, adhering to a modified procedure from the literature.^49^ In summary, 88 g of pentanol, 120 g of triethylamine, and 30 mL of dichloromethane (DCM) were introduced into a reaction flask maintained in an ice bath. Following this, 110 g of methanesulfonyl chloride (MsCl) was added gradually while continuously stirring. Once the addition was complete, the reaction mixture was allowed to reach room temperature and was stirred for an additional 15 min.

To eliminate any residual triethylamine, the reaction mixture underwent three washes with a 10% (w/v) aqueous citric acid solution, followed by three extractions with a 10% aqueous sodium bicarbonate (NaHCO_3_) solution. The organic phase was then collected, and the solvent was evaporated under reduced pressure.

The resulting intermediate was subsequently dissolved in 50 mL of acetonitrile (ACN), to which 0.8 equiv of 1,2-dimethylimidazole was added. The reaction mixture was heated to 60 °C and stirred for a duration of 12 h. Upon completion, the solvent was removed under a vacuum, and the crude product was extracted using hexane. The excess hexane was then evaporated, yielding a white solid product, which was characterized by nuclear magnetic resonance (NMR) spectroscopy to verify its structure.

The NMR spectrum of [C5DMIM]­[OMs] (200 MHz, CDCl3) exhibited signals at the following chemical shifts: 0.86–0.88 (3H, t), 1.86–1.91 (4H, m), 2.85 (3H, s), 3.85–3.87 (2H, t), 3.91 (3H, s), 4.12 (3H, s), 7.84–7.85 (1H, d), and 7.87–7.88 (1H, s).

### Ethics Statement

All animal experiments were performed in compliance with the Guidelines for the Ethics of Animal Experimentation established by the National Health Research Institutes in Taiwan. The experimental protocol received review and approval from the Institutional Animal Care and Use Committee at Taipei Medical University (Approval No. 112029-A-S01).

### Immunization of Mice

Outbred CD1 mice were utilized for the purpose of conducting immunization studies. Systemic immunization was achieved through intraperitoneal injection, with each experimental group comprising two mice. Each mouse received three doses of 0.1 mg of gliadin (extracted from wheat; Sigma-Aldrich) combined with 0.05 mg of muramyl dipeptide (MDP; Sigma-Aldrich) in a total volume of 0.2 mL of phosphate-buffered saline (PBS, pH 7.4). The injections were administered at intervals of 3 weeks.

Twelve hours following the final immunization, blood samples were collected and allowed to clot at 4 °C. Serum was subsequently separated via centrifugation at 15,000 × *g* for 20 min at room temperature and stored at–80 °C for future analysis. Flow cytometry and enzyme-linked immunosorbent assay (ELISA) were utilized to identify specific monoclonal antibody pairs (designated as E1 and E2) and to evaluate their binding affinity to gliadin. All immunization procedures were conducted in a specific pathogen-free (SPF) facility at Taipei Medical University, Taiwan. In this report, we assessed the purity of the antibody using SDS-PAGE analysis. The results from the SDS-PAGE revealed the presence of only two bands at 25 and 55 kDa, which correspond to the antibody, thereby indicating that the antibody achieved a purity level exceeding 99% following purification with Protein A. Furthermore, in the specificity evaluations conducted via ELISA for our self-developed antibodies, both E1 and E2 exhibited commendable specificity. Additionally, during the ELISA sensitivity assessment for gliadin detection, E1 demonstrated a superior detection limit of 10^–8^ mg/mL, in contrast to E2, which had a detection limit of 10^–6^ mg/mL, under identical experimental conditions. Data are shown in Figure S5.

### Preparation of the Lateral Flow Assay System

The lateral flow test strip was assembled by utilizing a sample pad, conjugate pad, nitrocellulose membrane, absorbent pad, and an adhesive backing card, with all components procured from commercial suppliers. For the establishment of the test (T) and control (C) lines, monoclonal antigliadin antibody E1 (60 μg/mL) and goat antimouse IgG (100 μg/mL; Sigma-Aldrich) were immobilized on the nitrocellulose membrane at designated test and control zones, respectively. Additionally, monoclonal antibody E2 was conjugated to gold nanoparticles (AuNPs) and subsequently applied to the conjugate pad. Prior to the application of the conjugate, the conjugate pad underwent treatment with a blocking buffer consisting of 5% bovine serum albumin (BSA), 0.5% Tween-20, 0.05% sodium azide, and 5% poly­(ethylene glycol) to mitigate nonspecific binding.

### Preparation of the AuNP-Conjugated Antibody

Gold nanoparticle (AuNP)–antibody conjugates were synthesized through the method of physical adsorption, which is a commonly employed technique for the immobilization of antibodies onto the surfaces of AuNPs.^47,48^ In this investigation, 10 μL of monoclonal antigliadin antibody (E2, 1 μg/mL) was combined with 1 mL of colloidal AuNP solution and incubated with gentle rotation at room temperature for a duration of 30 min to promote adsorption. Subsequently, bovine serum albumin (BSA) was introduced to block any unoccupied binding sites on the AuNP surface, and the mixture was incubated for an additional 15 min. The conjugates were then purified through centrifugation at 10,000 rpm for 30 min at 4 °C. This washing procedure was performed twice to ensure the complete removal of excess BSA. The resultant pellet was resuspended in a stabilization buffer containing 1% BSA and stored at 4 °C until further use.

### Sample Preparation for Gluten Detection

In order to isolate gluten from a range of food matrices, including wheat, chestnut, quinoa, oat, corn, chickpea, rice, and almond flour, a 75% (v/v) ethanol solution was employed as the extraction solvent. Specifically, each food sample was combined with the 75% ethanol and allowed to incubate at ambient temperature for a duration of 10 min. Following this incubation period, the resulting supernatant was subjected to filtration using a 0.22 μm syringe filter. Subsequently, the filtered extract was diluted in a 1:10 ratio with phosphate-buffered saline (PBS, pH 7.4) prior to further analyses.

### Preparation of Gliadin Standard Solutions

A standard stock solution was prepared by dissolving gliadin, which is derived from wheat, in 1 mL of 75% ethanol. The mixture was vortexed for 15 min to achieve homogeneity. Subsequently, the solution was filtered through a 0.22 μm syringe filter and then diluted with phosphate-buffered saline (PBS) to achieve a final gliadin concentration of 100 ppm. Serial dilutions were conducted by using PBS to create a standard curve with concentrations of 0, 3, 6, 9, 12, 25, 30, and 40 ppm. The gliadin concentrations were validated through an enzyme-linked immunosorbent assay (ELISA).

### Image Analysis System

A bespoke mobile application, named LeomyFood, was created to enhance the functionality of the LEO (Lateral flow Enhanced by Optical imaging) system. This application allows users to photograph lateral flow test strips, conduct automatic analyses of signal intensity, and ascertain gluten concentration levels. Additionally, it captures timestamps and geolocation metadata. All collected data are securely transmitted and stored in a cloud-based database, thereby enabling efficient remote monitoring and data management.

### LEO Assay Protocol

In the process of gluten detection utilizing the LEO system, a food sample weighing 20 mg was introduced into a specified extraction tube and mixed thoroughly. Subsequently, three drops of the resultant extract were deposited on the test strip. Following a two-minute incubation period, the test strip was photographed using the LeomyFood application, which subsequently delivered semiquantitative results within an additional minute.

### Preparation of Real Food Samples

Food samples were procured from various local sources, such as supermarkets, restaurants, and markets. For each analysis, a 20 mg portion of the food item was accurately measured, introduced into the LEO extraction tube, and thoroughly mixed. Subsequently, three drops of the resulting extract were deposited on the sensor chip. A discernible result was achieved within a two-minute time frame, and quantitative analysis was conducted utilizing the LeomyFood application.

### Statistical Analysis

All quantitative data are expressed as the mean ± standard deviation (SD). Statistical significance was assessed using a two-tailed *t*-test, with a *p*-value of less than 0.05 deemed statistically significant.

### Gliadin Solubility in Various Buffer Systems

In order to assess the extraction efficiency of various buffer systems for the recovery of gliadin, a sample of 1 g of wheat flour was combined with 10 mL of one of the following buffer solutions: phosphate-buffered saline (PBS), Tris buffer, carbonate buffer, or citrate buffer. The resulting mixtures were subjected to continuous stirring at ambient temperature for a duration of 12 h. Following the extraction process, the supernatant was obtained through centrifugation at 6000 rpm for 10 min at room temperature, and the gliadin content was subsequently analyzed utilizing the LEO assay system.

### Recovery Assessment of the LEO Assay

A recovery test was conducted using gluten-free rice noodles (Organic Rice Noodles, Yuan Shun Food Co.), which were verified to be gluten-free through ELISA analysis. Specifically, 40 g of the rice noodles was homogenized and subsequently spiked with 10 mL of a 10 ppm gliadin standard solution. Following this, the spiked samples underwent lyophilization to eliminate the solvent, and the remaining gluten content was quantified at room temperature by using the LEO gluten assay to evaluate the efficiency of gliadin recovery.

## Supplementary Material



## Notes

Zeta potential of different type AuNPs; competitive assay of iFAMs, 3M, GlutenTox and EZ gluten in test process and time for the gluten tester; competitive assay of iFAMs, rapid test and ELISA; cost of the gluten tester by general and new model system; statistical analysis of gluten test system; and characteristics of antibodies E1 and E2 (PDF)
