# Supplementary material for: Empowering Gliadin Detection: A Visible-Code Semiquantitative Lateral Flow System for Rapid and Reliable Results
Source: J Agric Food Chem. 2025 Sep 11;73(38):24369–78. doi: 10.1021/acs.jafc.5c07872 (PMC12464975; doi:10.1021/acs.jafc.5c07872)
Supplement: Supplementary file 1 [file jf5c07872_si_001.pdf]

Empowering Gliadin Detection: A Visible-Code Semi-quantitative Lateral Flow System for Rapid and Reliable Results

Wen-Hao Chen[ah+], Jill Christiansen Smith[a], Seaton Smith[a], Hui-Yin Huang[a], Chester Yuh-Cherng Chu[b], Yuen-Yee Choi[b], Yuyu Chen[b], Huan-Chi Chang[g], Chuan-Chih Hsu[cd*+], Yu-Cheng Hsiao[befg*]

[a] Research and Development Group, Leo Verification System Inc. Wyoming, US.

[b] Research and Development Group, Bion Inc., Taipei, Taiwan

[c] Department of Surgery, College of Medicine, Taipei Medical University, Taipei, Taiwan

[d] Department of Surgery, Taipei Medical University Hospital, Taipei, Taiwan

[e] Graduate Institute of Biomedical Optomechatronics, Taipei Medical University, Taipei, Taiwan

[f] Cell Physiology and Molecular Image Research Center, Taipei Medical University, Taipei, Taiwan

[g] uMeal Co., Ltd., Taipei 110, Taiwan

[h] Department of semiconductor Engineering, Lung-hwa University of Science and Technology

[+] These authors contributed equally to this work.

Nanoparticle type	Hydrodynamic Diameter (nm)	Zeta potential (mV)
AuNPs	32	-38.6
AuNPs-Ab	54	-16.7

Figure S1. Zeta potential of different type AuNPs.

Type of assay	Extraction	Time for testing	Digital assay	Reference
**iFAMs**	One-step	2 mins	Yes	-
**3M gluten protein rapid kit**	Two-Step	11 mins	No	40
**GlutenTox Sticks**	Two-step	15 mins	No	41
**EZ gluten**	Two-step	10 mins	No	42

Figure S2. The competitive assay of iFAMs, 3M, GlutenTox and EZ gluten in test process and time for gluten tester

	iFAMs	Rapid test	ELISA
Time-consuming	< 2 min	15 min	180 min
Convenience	Easy	Medium	Complicate
Device	No need	No Need	Expensive device
Quantify	Yes	No	Yes
Storage	Ambient	4℃	4℃
Analysis	Quantitative	Qualitative	Quantitative
Accuracy	99%	95%	98%

Figure S3. The competitive assay of iFAMs, Rapid test and ELISA.

Reagents	**Cost per assay**	
	General model	New model
Buffer	$ 1.1	$ 1.1
Antibody	$ 1.66	$ 1.66
Gluten	$0	$0.34
AuNPs	$ 0.6	$ 0.7
Strip	$ 1.2	$ 1.2
Total	$ 4.26	$ 5.0

Figure S4. Cost of gluten tester by general and new model system.

(A)

Sample	Repeat	Positive	Negative
0 ppm	30	0	30
3 ppm	30	3	27
5 ppm	30	28	2
10 ppm	30	30	0
15 ppm	30	30	0
20 ppm	30	30	0

(B)

Data from LEO system		Ture data		
		Positive	Negative	Total
	Positive	118	3	121
	Negative	2	57	59
	Total	120	60	180

(C)

PPM	T.P.	F.P.	T.N.	F.N.	Sensitive	Specific	F.P.%	F.N.%
5 ppm	118	3	57	2	98.3%	95%	2.5%	3.4%

Figure S5. Statistical analysis of gluten test system.

negative. Specificity:

Estimated specificity (Spec): 100%[T.P/(T.N+F.P)]

Sensitivity:

Estimated sensitivity (Sens): 100%[T.N/(T.P+F.N)]

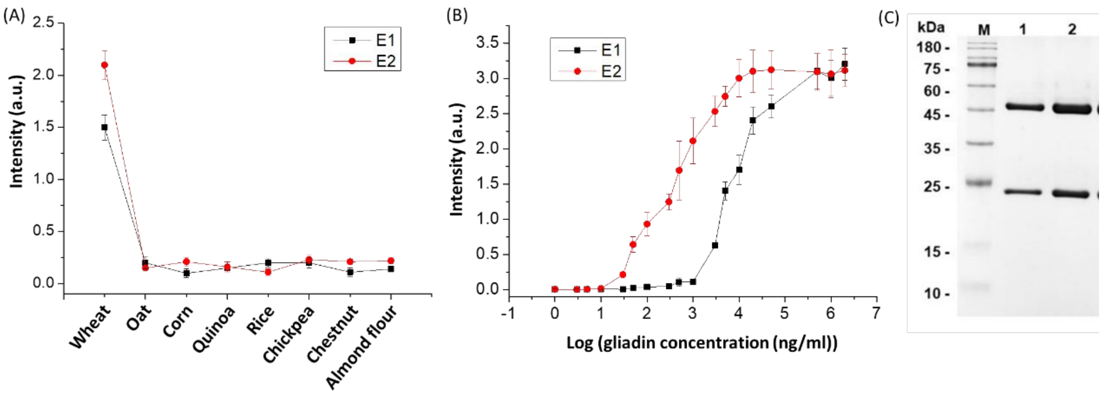

Figure S6. Characteristics of Antibodies E1 and E2

(A). Measure the specific levels of antibodies E1 and E2 in wheat and other cereal grains by ELISA.

(B). Measure the sensitivity of antibodies E1 and E2 at different concentrations of gliadin using the ELISA method.

(C). SDS-PAGE analysis of antibodies E1 (lane 1) and E2 (lane 2)
